# Clinical Diagnosis and Laboratory Testing of Abnormal Appearing Toenails: A Retrospective Assessment of Confirmatory Testing for Onychomycosis in the United States, 2022–2023

**DOI:** 10.3390/jof10020149

**Published:** 2024-02-13

**Authors:** Aditya K. Gupta, Tong Wang, Elizabeth A. Cooper, Sara A. Lincoln, Hui-Chen Foreman, William P. Scherer, Wayne L. Bakotic

**Affiliations:** 1 Division of Dermatology, Department of Medicine, University of Toronto, Toronto, ON M5S 3H2, Canada; 2 Mediprobe Research Inc., London, ON N5X 2P1, Canada; twang@mediproberesearch.com (T.W.); lcooper@mediproberesearch.com (E.A.C.); 3 Bako Diagnostics, Alpharetta, GA 30005, USA; slincoln@bakodx.com (S.A.L.); hforeman@bakodx.com (H.-C.F.); wscherer@bakodx.com (W.P.S.); wbakotic@bakodx.com (W.L.B.)

**Keywords:** onychomycosis, abnormal nail, nail disease, clinical diagnosis, confirmatory testing, fungal culture, histopathology, polymerase chain reaction, PCR

## Abstract

Onychomycosis is an under-recognized healthcare burden. Despite the risk of misdiagnosis, confirmatory laboratory testing is under-utilized. Histopathologic examination with polymerase chain reaction (PCR) is currently the most effective diagnostic method; it offers direct detection and identification of a fungal invasion. In this retrospective cohort study, we assessed confirmatory testing results, with matching clinical diagnoses, in 96,293 nail specimens submitted during a 9-month period from 2022 to 2023. Toenail specimens were examined using fungal culture, histopathology and/or PCR. Clinical diagnoses were identified using the International Classification of Diseases 10th Revision codes. For clinically diagnosed onychomycosis patients, the overall positivity rate was 59.4%; a similar positivity rate (59.5%) was found in patients with clinically diagnosed non-fungal nail dystrophy. Performing a histopathologic examination with PCR was more likely to provide pathogen identification results than using fungal culture. Male patients had a higher rate of onychomycosis overall; however, female patients had more non-dermatophyte mold onychomycosis caused by *Aspergillus*. Clinically diagnosed onychomycosis patients with a co-diagnosis of tinea pedis were more likely to test positive for onychomycosis by PCR (odds ratio [OR]: 4.2; 95% confidence interval [CI]: 2.7–6.4), histopathology (OR: 2.5; 95% CI: 2.0–3.1) and fungal culture (OR: 3.2; 95% CI: 1.5–6.6). Our results support the use of confirmatory laboratory testing when there is a clinical diagnosis of onychomycosis.

## 1. Introduction

Onychomycosis (tinea unguium) is a chronic superficial fungal infection characterized by nail dystrophies, including hyperkeratosis, brittleness, ridging and pitting, as well as nail discoloration and separation [1]. It is one of the most common nail disorders worldwide with a prevalence rate up to 17.7% [2]; this rate is up to 13.8% in North America [3,4,5] and 25.1% in Europe [6,7,8]. Despite common misconceptions, onychomycosis is not a self-limiting condition and is not merely a cosmetic concern; instead, this chronic condition can cause localized pain and discomfort resulting in abnormal gait, limited dexterity, psychological stress and impaired quality of life [9,10]. In immunocompromised individuals, this condition may result in systemic/deep infections [11,12].

Even though onychomycosis is a significant healthcare burden accounting for 12.7% of all outpatient visits by the elderly (≥ 65 years) in the United States [13], a recent study found that a significant portion of physicians do not prescribe treatment during the patient visit (78.9–82.0%) [14]. Consequentially, more than half of onychomycosis patients reported to have self-diagnosed and self-treated their condition, which likely contributed to a high treatment failure rate of 24.0% [15]. The current healthcare burden is further compounded by a high rate of misdiagnosis (25.5%) among general practitioners [16] and a growing number of patients seeking care from specialists rather than primary care providers [17].

Due to its clinical presentation, onychomycosis often requires differential diagnosis as it can mimic non-fungal nail disorders such as nail psoriasis, lichen planus, chronic microtrauma or subungual melanoma [1,18,19]. Although there are several clinical risk factors that can help physicians differentiate potential onychomycosis at the point-of-care [20,21,22], such as (a) toenail involvement, (b) male, (c) elderly, (d) diabetes, (e) peripheral vascular disease, (f) trauma and (g) concomitant tinea pedis, these observations alone do not always indicate onychomycosis with any degree of certainty; hence, it is recommended to conduct confirmatory laboratory testing prior to treatment initiation. Traditional methods of testing for onychomycosis include KOH direct microscopy, fungal culture and histopathologic examination, while emerging diagnostic modalities utilizing techniques such as polymerase chain reaction (PCR) offer promising potential in improving diagnostic sensitivity and turnaround time [23].

To better inform physicians about the utility of confirmatory testing, we examined records of 96,293 diagnostic nail samples submitted over a 9-month period from 2022 to 2023. All samples were tested using either fungal culture, histopathology, multiplex real-time PCR or any combination of the three per physician’s order. Confirmatory testing results were matched with clinical diagnoses of onychomycosis or other nail disorders. 

## 2. Materials and Methods

In this retrospective cross-sectional cohort study, we reviewed records of 96,293 toenail samples sent for confirmatory testing at one CLIA-certified molecular diagnosis laboratory from 29 November 2022 to 25 August 2023. Samples were submitted by dermatology and podiatry outpatient clinics across the U.S. (one sample per patient) with de-identified patient demographic information (i.e., sex and age), clinic location and clinical diagnosis per the International Classification of Diseases 10th Revision (ICD–10) coding system. Clinically diagnosed onychomycosis patients were identified using the ICD–10 code B35.1. Additional ICD–10 codes employed to stratify patient confirmatory testing results included tinea pedis (ICD–10 B35.3) and nail dystrophy (ICD–10 L60.3). All samples were provided by a qualified medical diagnosis laboratory as part of a non-interventional standard-of-care procedure. We reviewed secondary de-identified data only; as such, the current work does not represent a clinical trial for which ethics overview and informed consent was required.

Confirmatory testing was conducted using fungal culture, histopathologic examination and/or multiplex real-time PCR; each test was conducted following a previously described protocol [24]. The choice of test was made according to physician’s order, which includes the options of conducting a single test (e.g., histopathologic examination) or a combined test (e.g., histopathologic examination and PCR). 

### 2.1. Fungal Culture

Isolates were obtained and identified as previously described [24]. Briefly, nail specimens were cultured on potato dextrose agar and incubated for 2–4 weeks at 30 °C in ambient air. If the identification result was inconclusive, a subsequent mass spectrometry analysis (VITEK MS system; bioMérieux, Inc., Durham, NC, USA) was performed. 

### 2.2. Histopathology 

Histopathologic examination was carried out using a Periodic acid–Schiff reaction (PAS) and/or Grocott’s methenamine silver (GMS) stain per physician’s order. All fixed sample slides were examined by a dermatopathologist, as previously described [24]. Diagnostic parameters include (a) presence of fungal hyphae or yeast, (b) estimation of fungal element quantity (i.e., rare, minimal [<10%], moderate [10–80%] or florid [>80%]), (c) infection pattern (i.e., subungual, superficial or total dystrophic) and (d) additional pathologic findings (e.g., onycholysis). 

### 2.3. Multiplex Real-Time PCR

Nail specimens were treated with 1 mL of lysis buffer in beaded tubes (Omni International, Kennesaw, GA, USA) and homogenized in three steps: (1) 5-min homogenization on a bead ruptor (Omni International, Kennesaw, GA, USA), then (2) 10-min incubation at 90 °C in a dry bath and (3) 2.5-min centrifugation at a speed of 12,500 rpm [24]. DNA extraction from homogenized samples was performed on an automated workstation (Hamilton Microlab STAR) using a magnetic bead-based separation method (Mag–Bind Plant DNA DS Kit [Omega Biotek, Norcross, GA, USA]).

Fungal detection and identification from DNA extracts were performed using the BakoDx Onychodystrophy Agent Detection (OIAD) assay (Bako Diagnostics, Alpharetta, GA, USA), a multiplex real-time PCR assay with two sequential panels [24]. Detection and identification targets include dermatophytes (*Trichophyton mentagrophytes* complex, *T. rubrum* complex, *Epidermophyton* spp., *Microsporum* spp.), non-dermatophyte molds (NDMs; *Acremonium* spp., *Alternaria* spp., *Aspergillus* spp., *Curvularia* spp., *Fusarium* spp., *Scopulariopsis* spp., *Neoscytalidium* spp.) and yeasts (*Candida albicans*, *C. guilliermondii*, *C. parapsilosis*, *C. tropicalis*, *Cryptococcus* spp., *Malassezia* spp., *Trichosporon* spp.). 

### 2.4. Data Analysis

Confirmatory testing results were dichotomized into positive or negative categories per use of fungal culture, histopathology or PCR. For samples that were tested using a combined method (e.g., histopathologic examination and PCR), a positive result is presented as double positives (e.g., PAS/GMS+/PCR+ or PAS/GMS+/Culture+) or single positive (e.g., PAS/GMS+, PCR+ or Culture+). Results were further stratified by patient characteristics; patients with missing demographic data were excluded from sub-analysis (age: 0.02% [18/96293]; sex: 2.3% [2254/96293]; U.S. state: 0.02% [18/96293]). 

Statistical significance was determined using the chi-square test and two-sided two proportion Z test. Effect estimates were calculated using the odds ratio (OR) and 95% confidence interval (CI); two-sided *p*-values were calculated as previously described by Altman and Bland [25]. All analyses were carried out using the Excel^®^ software package (version 2301), with an alpha level of 0.05.

## 3. Results

A total of 96,293 nail samples were received during the 9-month period from November 2022 to August 2023. The mean (SD) age of this cohort was 56.7 (18.5), with 61.4% (57735/94039) females. The majority of samples were submitted from the Southern U.S. region (44.4% [42731/96275]). Clinically suspected onychomycosis cases diagnosed by physicians at the point-of-care accounted for 51.5% (49581/96293) of samples, based on submitted clinical information identified by the ICD–10 code B35.1. A nail dystrophy diagnosis (ICD–10 code L60.3) was identified in 38.5% (37026/96293) of samples, and a tinea pedis diagnosis (ICD–10 code B35.3) was identified in 1.0% (957/96293) of samples. Most samples were tested by histopathologic examination only (47.6% [45826/96293]) or combined histopathologic examination with multiplex real-time PCR (42.9% [41284/96293]); in contrast, only 0.3% (278/96293) of samples were tested using only fungal culture.

**Figure 1:**
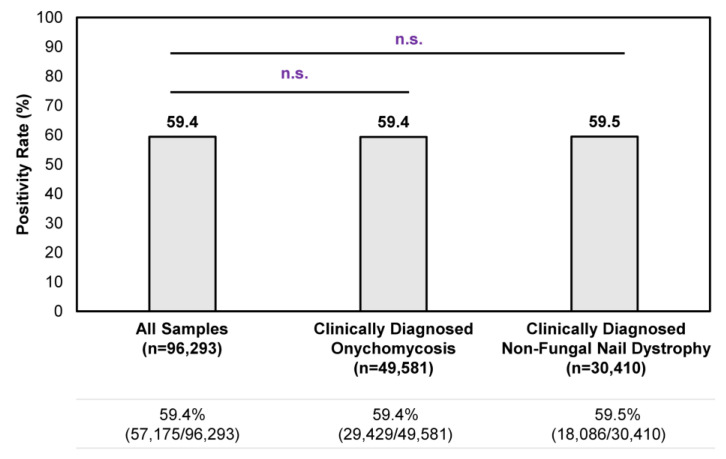
Overall confirmatory testing positivity rates among samples received during the 9-month study period. A total of 96,293 samples were tested during the study period, of which 49,581 unique samples were identified from patients with a clinical diagnosis of onychomycosis (i.e., samples identified with ICD–10 code B35.1) and 30,410 unique samples were identified from patients with a clinical diagnosis of non-fungal nail dystrophy (i.e., samples identified with ICD–10 code L60.3 without B35.1). n.s.—not significant.

Overall, a consistent positivity rate was observed regardless of clinical suspicion of onychomycosis or non-fungal nail dystrophy (Figure 1). Among all samples tested during the study period, the positivity rate was 59.4% (57,175/96,293); no statistically significant differences in positivity rate were observed for clinically diagnosed onychomycosis cases or non-fungal nail dystrophy cases. In other words, the clinical presentation of onychomycosis is often ambiguous to healthcare providers, as reflected by the 59.5% (18,086/30,410) positivity rate in samples with a clinical diagnosis of nail dystrophy of non-fungal origin (i.e., samples identified by ICD–10 code L60.3 without B35.1). These findings further strengthen the need to perform confirmatory testing for the appropriate management of onychomycosis.

### 3.1. Geographical Distribution of Abnormal Appearing Toenails Submitted for Confirmatory Testing of Onychomycosis

**Figure 2:**
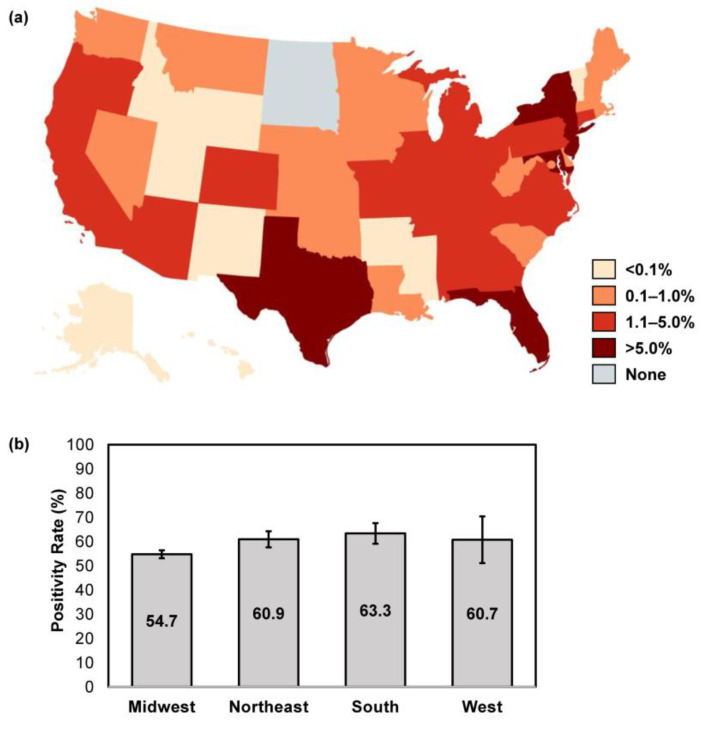
Geographic differences in sample size and overall positivity rates. (**a**) The proportion of abnormal appearing toenail specimens received over the study period were stratified per U.S. state; image created using MapChart. (**b**) The overall positivity rate was calculated for each respective U.S. census region; error bars represent 95% CI.

Location data were available for 96,275/96,293 samples (99.98%) received over the study period. The majority of samples were submitted from the Southern U.S. region (44.4% [42,731/96,275]), followed by the Northeast (31.5% [30,336/96,275]), Midwest (15.1% [14,555/96,275]) and West (8.9% [8546/96,275]). States with the highest volume of sample submission for confirmatory testing were New York (13.9% [13,341/96,175]), New Jersey (10.6% [10,172/96,175]), Florida (9.6% [9192/96,175]), Maryland (7.8% [7515/96,175]) and Texas (7.7% [7422/96,175]) (Figure 2a). The overall confirmatory testing positivity rate was similar across all U.S. regions, at 54.7–63.3% (Northeast: 60.9% [95% CI: 57.5–64.2]; Midwest: 54.7% [95% CI: 53.1–56.3]; South: 63.3% [95% CI: 59.1–67.6]; West: 60.7% [95% CI: 51.0–70.3]) (Figure 2b).

### 3.2. Confirmatory Testing per Utilization of Fungal Culture, Histopathology, PCR or Combination

**Figure 3:**
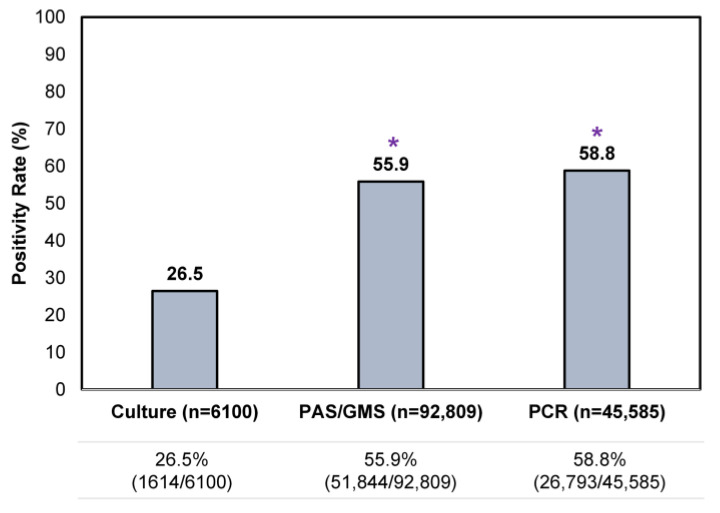
Comparison of single confirmatory testing results—per utilization of histopathology (PAS/GMS), PCR or culture—in all samples received during the study period. * *p* < 0.05 compared to culture.

An assessment of all testing results showed that a single culture result was the least likely to be positive (26.5% [1614/6100]) compared to a single histopathologic exam or PCR test (Figure 3), reflective of its lower sensitivity. Positivity rates for a single histopathologic exam and PCR were similar, at 55.9–58.8%.

**Figure 4:**
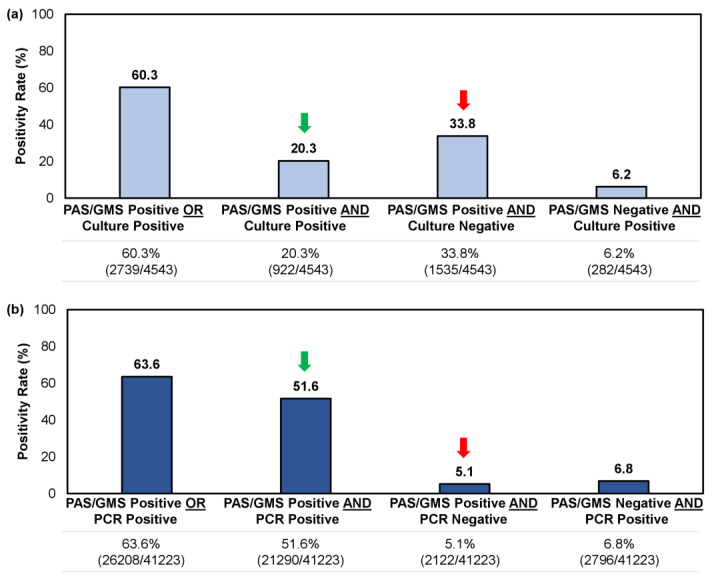
Comparison of confirmatory testing results per utilization of (**a**) a combined histopathologic exam with culture or (**b**) a combined histopathological exam with PCR. Results were further stratified into single positive or double positive results. Green arrow indicates the rate of double positive samples (i.e., PAS/GMS+/Culture+ or PAS/GMS+/PCR+); red arrow indicates the rate of single positive samples per histopathologic exam but negative on culture or PCR (i.e., PAS/GMS+/Culture- or PAS/GMS+/PCR-).

For samples tested using a combined histopathologic exam with culture or PCR, we reviewed 4543 and 41,223 unique records, respectively. Both methodologies yielded a similar positivity rate overall (i.e., a single positive result [PAS/GMS+/Culture-, PAS/GMS+/PCR-, PAS/GMS-/Culture+, PAS/GMS-/PCR+] or double positive result [PAS/GMS+/Culture+ or PAS/GMS+/PCR+]), at 60.3–63.6% (Figure 4); however, the likelihood of obtaining a double positive sample—providing direct indication of a fungal invasion while identifying the pathogen—was significantly higher when a histopathologic examination (PAS/GMS) was performed in combination with PCR rather than culture (51.6% [21,290/41,223] vs. 20.3% [922/4543]; *p* < 0.05). Similarly, the likelihood of obtaining a single positive result from histopathology, without corroboration through identifying the causative fungal agent, was greater for the culture method than the PCR method (33.8% [1535/4543] vs. 5.1% [2122/41,223]; *p* < 0.05) (Figure 4).

### 3.3. Confirmatory Testing in Different Age Groups

**Figure 5:**
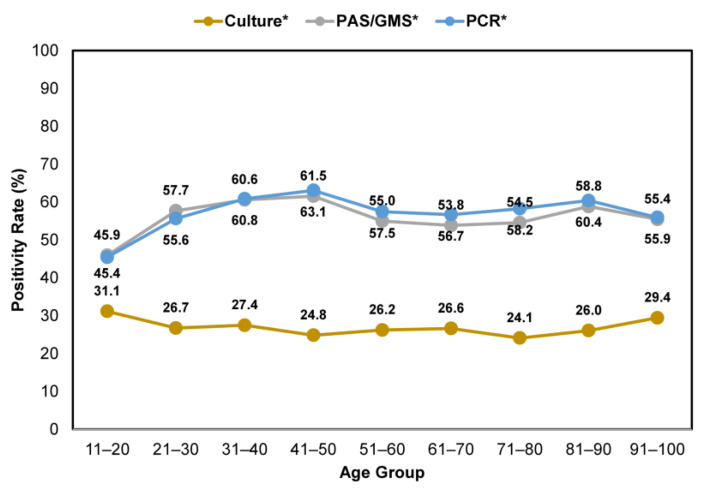
Comparison of confirmatory testing positivity rates—per utilization of histopathology (PAS/GMS), PCR or culture—in patients with clinically apparent onychomycosis across age groups. * Including samples that were tested using a single and combined method.

Across all age groups, nail specimens that were tested using either histopathology or PCR consistently showed higher positivity rates than fungal culture (Figure 5). The positivity rates varied between 45.9–61.5% and 45.4–63.1% for clinically suspected onychomycosis cases tested by histopathology and PCR, respectively. In contrast, lower positivity rates, between 24.1 and 31.1%, were observed for samples tested using the fungal culture method.

### 3.4. Distribution of Fungi Groups in PCR-Positive Samples with or without Corroboration by Histopathology

A drawback of PCR diagnosis is the potential inability to differentiate ‘true’ pathogens from commensal organisms and environmental contaminants, which can thus lead to false positive results. Hence, combining the use of PCR diagnosis with histopathologic examination, especially in the case of suspected NDM onychomycosis, has been suggested as a reliable proxy for true infection with the demonstration of nail plate invasion [21,24]. For samples tested using combined PCR and histopathology (*n* = 41,284), we assessed the relative proportions of dermatophytes, NDMs, yeasts and mixed detections in PCR-positive and histopathology-positive samples (51.6% [21,290/41,284]), compared to PCR-positive and histopathology-negative samples (6.8% [2796/41,284]).

In PCR-positive and histopathology-positive samples, NDM and yeasts were detected at 19.3% (4115/21,290) and 5.6% (1182/21,290), respectively. In comparison, NDMs (34.7% [970/2796]) and yeasts (25.6% [715/2796]) observed in PCR-positive and histopathology-negative samples likely reflects the absence of nail plate invasion and could be construed as a potential false positive result requiring re-assessment. The detection of dermatophytes (22.7% [635/2796]) by PCR but not histopathology could be partially attributed to sampling bias, where fungal elements were absent in the portion of the nail specimen submitted for testing. With dermatophytic fungi causing the majority of true positive onychomycosis cases, these positive PCR results, in conjunction with negative histopathology, likely represent true positive results.

### 3.5. Confirmatory Testing and Fungi Detection/Identification Pattern in Male and Female Onychomycosis Patients 

Male patients were more likely to test positive for onychomycosis than female patients by confirmatory testing, especially by histopathology (67.2% [12,324/18,352] in males vs. 48.9% [14,134/28,900] in females) and PCR (69.2% [5922/8540] in males vs. 50.6% [6631/13,109] in females). 

**Figure 6:**
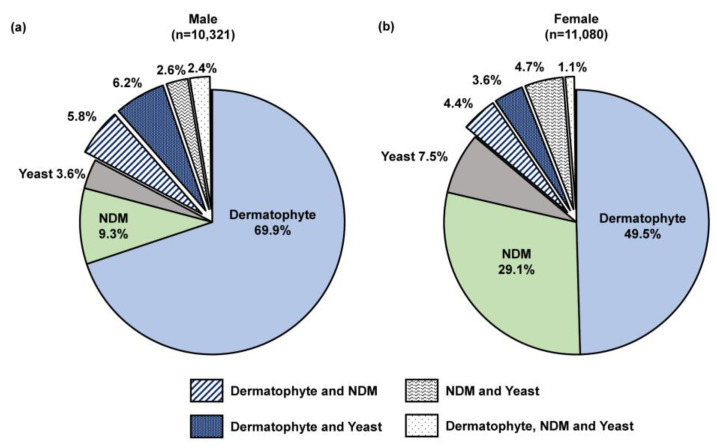
Fungi group distribution in (**a**) 10,321 male and (**b**) 11,080 female onychomycosis patients; all patients were PCR-positive and histopathology-positive, 0.2% of samples from both males (16/10,321) and females (21/11,080) were unidentified likely due to the organism being outside of the assay detection range or insufficient starting material. NDM—non-dermatophyte mold.

**Figure 7:**
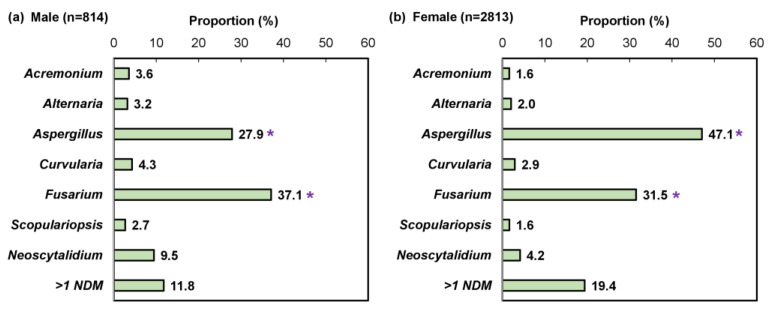
Pathogen identification pattern in (**a**) 814 male and (**b**) 2813 female non-dermatophyte mold onychomycosis patients; mixed infections (e.g., dermatophyte and non-dermatophyte mold) were excluded. * *p* < 0.05 compared to the opposite sex.

We performed a sub-analysis of samples testing positive by combined histopathologic examination with PCR (*n* = 21,823), which decreases the likelihood of false positive results while enabling pathogen differentiation as described previously [24]. A significantly higher proportion of female patients were detected with an NDM pathogen compared to male patients (29.1% [3222/11,080] vs. 9.3% [957/10,321]; *p* < 0.05; Figure 6). Furthermore, a significant difference was observed in the NDM identification results (Figure 7). For both sexes, *Aspergillus* and *Fusarium* were the most common fungal genera; however, *Aspergillus* was significantly more common in female patients than male patients (47.1% [1324/2813] vs. 27.9% [227/814]; *p* < 0.05).

In agreement with previous findings, dermatophytes were detected as the predominant pathogen for both sexes (male: 69.9% [7211/10,321], female: 49.6% [5490/11,080]). The *T. rubrum* complex was detected as the most common dermatophyte (male: 90.0% [6333/7035], female: 86.2% [4617/5355]), followed by the *T. mentagrophytes* complex (male: 8.2% [575/7035], female: 12.8% [687/5355]). No significant difference in the identification pattern between the sexes was found for dermatophytes and yeasts.

### 3.6. Confirmatory Testing in Clinically Apparent Onychomycosis with Co-Diagnoses

**Figure 8:**
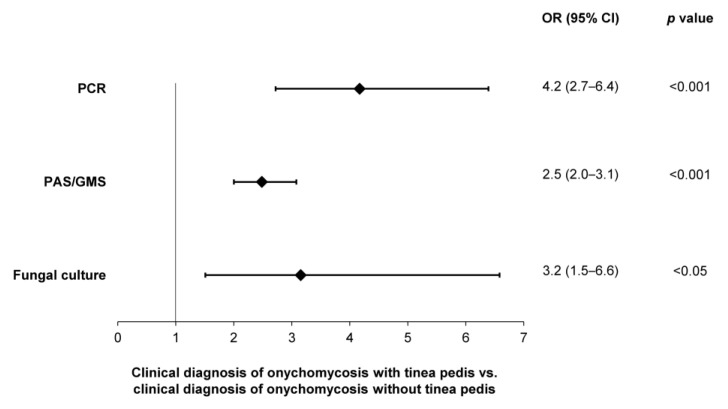
Likelihoods of obtaining a positive sample—per utilization of PCR, histopathology (PAS/GMS) or fungal culture—in patients with clinical evidence of onychomycosis and tinea pedis.

A significantly higher likelihood of testing positive—per PCR (OR: 4.2; 95% CI: 2.7–6.4), histopathology (OR: 2.5; 95% CI: 2.0–3.1) and fungal culture (OR: 3.2; 95% CI: 1.5–6.6)—was observed in patients with a clinical diagnosis of onychomycosis and tinea pedis, compared to onychomycosis patients without tinea pedis (Figure 8). In contrast, no difference in the likelihood of testing positive was observed in patients with a clinical diagnosis of onychomycosis and nail dystrophy.

## 4. Discussion

An accurate diagnosis of onychomycosis requires confirmatory laboratory testing, in addition to a thorough physical examination. Out of the available techniques, the most effective testing method is a combined histopathologic examination, where PCR testing as the first test indicates evidence of a fungal invasion in the nail plate and the second test provides pathogen identification. This combination was also recommended as the most effective diagnostic procedure by the German S1 guideline—a guideline reflecting the recommendations of professional medical associations in the field of dermatology and mycology [21]. Due to the distinct possibility of NDMs being present in nails as environmental contaminants or commensal organisms [26], performing a histopathologic examination with PCR not only improves the diagnostic accuracy but also greatly shortens the time-to-treatment initiation, as the traditional fungal culture method requires ≥2 sequential isolations spaced at least 1 week apart [24,27]. In addition to its long turnaround time and low sensitivity, the traditional culture method is also subject to a higher risk of false negative results, especially in patients who were recently treated with antifungals [28]. 

In this study, we analyzed results of 96,293 nail samples sent for confirmatory testing and found a congruent positivity rate of 59.4–59.5%, irrespective of whether patients had been clinically diagnosed with onychomycosis or non-fungal nail dystrophy (Figure 1). This finding further corroborates the general consensus that only about half of the clinically suspected cases of onychomycosis are proven positive by laboratory testing and reinforces our viewpoint that clinical diagnosis of onychomycosis is not sufficient as a solitary diagnostic approach; laboratory testing must be performed.

Our analysis of the sample submission pattern has identified several U.S. regions with potentially higher disease burdens (Figure 2a). The differences observed across U.S. census regions was congruent with the results of a 2018 study [13]; in particular, the higher proportion of abnormal appearing toenail samples from the Southern U.S. region can be explained by the higher number of elderly residents predisposed to a higher risk of infection and a warmer/more humid climate favoring fungal growth [20,22]. In areas with high population densities, person-to-person spread of onychomycosis can present an additional challenge to healthcare providers amidst concerns of antifungal resistance. In a recent study by Hwang et al. [29], 43.8% (28/64) of onychomycosis patients seen at a dermatology center in New York (2022–2023) failed to respond to oral terbinafine treatment; of these, terbinafine resistance mutations were confirmed in 11.7% (2/17) of cases. 

When deciding which testing modality to use in the confirmation of onychomycosis, it is important to consider qualitative differences between each method, in addition to sensitivity and specificity. In this study, we found that histopathologic examination and multiplex real-time PCR exhibited higher positivity rates than the traditional culture method (Figure 3); furthermore, this marked difference was consistent across all patient age groups suspected of onychomycosis (Figure 5). Performing a histopathologic examination with fungal culture is less likely to provide accurate pathogen identification results compared to histopathologic examination with PCR, which limits its clinical utility (Figure 4). However, performing a single PCR test may inadvertently detect environmental contaminants or commensal organisms unrelated to nail pathology, as evidenced by the higher proportions of NDMs and yeasts found in PCR-positive/histopathology-negative samples; albeit the extent of this impact on diagnostic accuracy is uncertain as it only occurred in up to 6.8% of samples. Histopathology is considered a cost-effective method of providing direct evidence of fungal invasion in the nail [30]; this helps to put into context the potential false positive findings that may be associated with using PCR testing alone. By contrast, performing histopathologic examination alone does not provide information on the identity of the specific pathogen, which is important in the development of an appropriate treatment plan [30]. 

In agreement with previous findings, we found that samples collected from male patients were more likely to test positive, with a higher proportion of dermatophytes than in samples from female patients [2,3]. Furthermore, we found that female patients suspected of having onychomycosis were more likely to have an infection with NDMs (Figure 6); pathogen identification results indicated a higher prevalence of *Aspergillus* compared to male patients (Figure 7). In previous studies, the emergence of NDM onychomycosis has been reported using culture-based methods without repeated sampling [31,32] and the association of female patients with NDM pathogens, especially *Aspergillus*, has been reported sparingly, mostly outside of the U.S. [33,34,35,36,37,38]. In this study, we confirmed a differential pathogen profile in male and female onychomycosis patients using combined histopathologic examination with multiplex qPCR. Considering that some of the female NDM onychomycosis patients reported previously were young adults without underlying co-morbidities [33,34], the higher propensity for NDM infections in this patient group could partially be attributed to a higher use of open-toed footwear and nail (micro)trauma due to more frequent nail trimming or use of ill-fitting footwear; however, further studies are warranted to confirm this observation.

Clinically apparent onychomycosis patients presenting with tinea pedis were 2.5–4.2 times more likely to test positive with a mycological diagnosis of onychomycosis than those without a co-diagnosis of tinea pedis (Figure 8). With tinea pedis being a well-recognized risk factor associated with onychomycosis [20,39,40], in these cases, tinea pedis may precede onychomycosis and likely represents the initial site of infection. An increased awareness of clinical risk factors may assist physicians in improving their diagnostic confidence and initiating treatments as appropriate. 

This study is limited by the retrospective design and use of results that were obtained from a single medical diagnosis laboratory; hence, our findings may not be generalizable to the entire patient population. With the advent of newer molecular diagnostic procedures, further prospective surveillance studies are warranted to better inform physicians about the at-risk populations and the pathogen spectrum, thereby enabling personalized onychomycosis management. The negativity rate for fungal culture observed in this study (73.5%) was higher compared to a recent report (51.1%); this difference may be attributed to different sampling methods and the type of specimen collected [41]. 

Current gaps in onychomycosis management practices expose patients to higher risks of misdiagnosis and mistreatment. Healthcare providers treating patients who are clinically suspected of having onychomycosis should consider submitting nail samples for confirmatory laboratory testing prior to initiating treatment; doing so would not only exclude the possibility of other common nail disorders and mitigate risks associated with systemic agents, but also increase the likelihood of a positive clinical response and higher patient satisfaction. 

## 5. Conclusions

The diagnosis and treatment of onychomycosis, especially for NDMs and mixed infections, will likely remain an ongoing challenge. Obtaining an accurate and precise diagnosis of onychomycosis requires confirmatory testing in addition to physical examination. A greater likelihood of obtaining a mycology-confirmed diagnosis can be achieved by recognizing established risk factors at clinical presentation (i.e., differential diagnosis); for onychomycosis, our findings from this retrospective analysis of 96,293 toenail specimens resonated with previous studies that found male patients and patients with a co-diagnosis of tinea pedis to be more at-risk. Furthermore, female patients exhibited higher propensities for NDM infections, especially by *Aspergillus*. By utilizing all three currently available methods for mycology testing—fungal culture, histopathology and PCR—the present study found that, with or without clinical evidence of fungal infection, half of the nail samples submitted for confirmatory testing were positive for onychomycosis, thus further highlighting the similarities among the clinical manifestations of the various etiologies of onychodystrophy, both infectious and non-infectious. Performing a combination of histopathologic examination and PCR testing has the greatest clinical utility as this approach allows for the demonstration of nail keratin invasion, thus representing a ‘true positive’ result while also identifying the causative agent of disease. Performing only a PCR diagnosis, without corroboration by histopathology, may carry a higher risk of detecting NDMs and yeasts without evidence of nail invasion or ‘true infection’. By contrast, performing only a histopathologic examination is not able to provide genus- or species-level identification, and thus does not allow for more targeted treatment approaches. Amidst the current high risk of misdiagnosis and mistreatment of onychomycosis [13,15,16], a greater advocacy for laboratory testing is warranted to address these pressing challenges. 

## Data Availability

Data are contained within the article.
